# The diagnosis of inherited metabolic diseases by microarray gene expression profiling

**DOI:** 10.1186/1750-1172-5-34

**Published:** 2010-12-01

**Authors:** Monica Arenas Hernandez, Reiner Schulz, Tracy Chaplin, Bryan D Young, David Perrett, Michael P Champion, Jan-Willem Taanman, Anthony Fensom, Anthony M Marinaki

**Affiliations:** 1 Purine Research Laboratory. GSTS Pathology. Guy's and St. Thomas' Hospitals, London, UK; 2 Division of Medical and Molecular Genetics. King's College London, London, UK; 3 Cancer Research UK Medical Oncology Laboratory. Barts and the London School of Medicine and Dentistry. Queen Mary, University of London, London, UK; 4 Genetics Centre. GSTS Pathology. Guy's and St. Thomas' Hospitals, London, UK; 5 Institute of Cancer, Barts and the London School of Medicine, Queen Mary University, London, UK; 6 Chemistry Department. Barts and the London School of Medicine and Dentistry. Queen Mary, University of London, London, UK; 7 Department of Inherited Metabolic Disease. Evelina Children's Hospital, London, UK; 8 Department of Clinical Neurosciences. Institute of Neurology, University College London, UK

## Abstract

**Background:**

Inherited metabolic diseases (IMDs) comprise a diverse group of generally progressive genetic metabolic disorders of variable clinical presentations and severity. We have undertaken a study using microarray gene expression profiling of cultured fibroblasts to investigate 68 patients with a broad range of suspected metabolic disorders, including defects of lysosomal, mitochondrial, peroxisomal, fatty acid, carbohydrate, amino acid, molybdenum cofactor, and purine and pyrimidine metabolism. We aimed to define gene expression signatures characteristic of defective metabolic pathways.

**Methods:**

Total mRNA extracted from cultured fibroblast cell lines was hybridized to Affymetrix U133 Plus 2.0 arrays. Expression data was analyzed for the presence of a gene expression signature characteristic of an inherited metabolic disorder and for genes expressing significantly decreased levels of mRNA.

**Results:**

No characteristic signatures were found. However, in 16% of cases, disease-associated nonsense and frameshift mutations generating premature termination codons resulted in significantly decreased mRNA expression of the defective gene. The microarray assay detected these changes with high sensitivity and specificity.

**Conclusion:**

In patients with a suspected familial metabolic disorder where initial screening tests have proven uninformative, microarray gene expression profiling may contribute significantly to the identification of the genetic defect, shortcutting the diagnostic cascade.

## Background

At least 300 different IMDs have been described [1] and new disorders are being identified [2,3] due to increasing awareness and advances in identification techniques. The birth prevalence of IMDs in the West Midlands is estimated to be 1 in 784 live births, extrapolating to approximately 800 new cases per year in the UK as a whole [4]. The majority of patients (72%) are diagnosed by the age of 15 years, with only one-third diagnosed by the age of one year. Any hope of effective treatment rests on precise and early diagnosis [4,5]. The diagnosis of IMDs may be a long and tedious process. The first step relies on matching clinical presentation to a potentially defective metabolic pathway. These investigations may take several months to complete, and even after this time, it may not be possible to make a diagnosis. Indeed, our experience in the Purine Research Laboratory at Guy's and St Thomas' Hospitals shows that a definitive diagnosis is only made in about 1% of children investigated for a suspected purine or pyrimidine disorder, with one reason being the overlap in clinical presentation between unrelated metabolic disorders. In the majority of cases, referrals are made for purposes of disease exclusion, or as part of a differential diagnosis.

We have undertaken a study using microarray gene expression profiling of cultured fibroblasts to investigate patients with a broad range of suspected metabolic disorders, including defects of lysosomal, mitochondrial, peroxisomal, fatty acid oxidation, carbohydrate, amino acid, molybdenum cofactor, and purine or pyrimidine metabolism (Table 1). The aim of the study was to define a gene expression signature characteristic of a defective metabolic pathway. No characteristic transcriptome-wide signatures were evident. However, we found that in 16% of cases the defective gene could be identified from the gene expression data irrespective of the underlying metabolic disorder.

**Table 1:** Inherited metabolic disorders included in this study and number of patients.

Disorder	Num of patients
	N = 68
**Lysosomal storage disorders**	
Niemann Pick A, B, C	7
Gaucher disease	1
Tay-Sachs disease	2
Cystinosis	1
Batten's disease	1
Aspartylglucosaminuria	1
Fabry's disease	1
Farber's disease	1

**Purine and Pyrimidine disorders**	
Lesch-Nyham disease/HPRT deficiency	3
Purine nucleotidase (PNP) deficiency	2
Adenylosuccinate lyase (ADSL) deficiency	1
Adenosine deaminase (ADA) deficiency	1
Dihydropyrimidine dehydrogenase (DPD) deficiency	2
**Peroxisomal disorders**	

Zellweger disease	4
Adrenoleukodystrophy	2
Rhizomelia chondrodisplasia punctata	1
**Urea cycle defect**	
Argininosuccinic aciduria	2

**Fatty acid oxidation disorders**	
Carnitine transport defect	2
Short-chain acyl-CoA dehydrogenase (SCAD) deficiency	1
Medium-chain acyl-CoA dehydrogenase (MCAD) deficiency	2
Very long-Chain acyl-CoA dehydrogenase (VLCAD) deficiency	1

**Mitochondrial disorders**	
Deoxy-guanosine kinase (DGUOK) deficiency	1
Surfeit-1 (SURF1) deficiency	1
Polymerase DNA-directed gamma (POLG) deficiency	3
Lactic acidosis	1

**Carbohydrate metabolism**	
Glycerol kinase (GK) deficiency	1
Pompe disease	2

**Others**	
Molybdenum cofactor deficiency	2
Isolated sulphite oxidase deficiency	1
**Unknown disorders**	14
**Non-affected**	3

## Methods

### Patient samples and tissue culture

Human skin fibroblast cell lines from 68 patients with suspected or confirmed metabolic disorders (Table 1) were recovered from the cell bank held by the Enzyme Laboratory, Medical and Molecular Genetics, Guy's Hospital. Cells were cultured with Ham's F10 medium supplemented with 10% foetal bovine serum, 2% L-glutamine (200 mM), 2% penicillin (5.0 IU/ml) and streptomycin (5.0 μg/ml) at 37°C in a closed system. Passage numbers were recorded where known.

Cell lines were screened for *Mycoplasma *infection using Venor^®^GeM *mycoplasma *detection kit for conventional PCR (Minerva Biolabs GmbH, Germany).

### RNA extraction and microarrays

Cells were grown in triplicate to sub-confluence. Total RNA from triplicate flasks was extracted using the RNeasy^® ^Mini kit™(QIAGEN, Crawley, UK). The pooled RNA was then concentrated using RNeasy^® ^MinElute™Cleanup kit (QIAGEN) and quantified by spectrophotometric analysis measuring absorbance at 260 and 280 nm. Double stranded cDNA was synthesised from 5 μg RNA using the Affymetrix One-cycle cDNA synthesis kit following the manufacturer's instructions (Affymetrix, High Wycombe, UK). Synthesis of Biotin-Labelled cRNA was performed using the Affymetrix GeneChip IVT Labelling kit, following the manufacturer's instructions. Labelled cRNA was then purified (sample cleanup module) and fragmented and 15 μg hybridized to Affymetrix GeneChip^® ^Human Genome U133 Plus 2.0 arrays overnight.

### Analysis of mycroarray data

Probe level summarization of all arrays was performed twice using two different methods: Robust multiarray averaging [6] (RMA) and Factor analysis for robust microarray summarization [7] (FARMS). In addition, Informative/Non-Informative (I/NI) P-values were computed [8]. Control probe sets and probe sets with a relatively large number of non-aligning probes or non-uniquely aligning probes were excluded. Inclusion criteria for a probe set were that 7 or more probes (out of a total of 11 for most probe sets) had to perfectly match the human transcriptome, and the median number of perfect matches per probe had to be less than 1.5 for a probe set to be included. In the case of the RMA-summarized data, a probe set had to also exceed a median expression level of 100 (linear scale) across all arrays, resulting in 11,753 probe sets entering into the subsequent analyses. In the FARMS case, only informative probe sets were considered (I/NI P-value of less than 0.6), leaving a total of 9,787 probe sets for analysis. We refer to the measurements taken by the included probe sets for a patient sample as the sample's expression profile. Principal component analysis (PCA) was applied to identify and quantify independent sources for the variance observed in the data. Matlab r2007a was used for correlation, hierarchical clustering and PCA.

We used two metrics to determine the degree to which a gene expression measurement x constitutes an outlier: Dixon's Q statistic defined as (2nd-to-minimal-value-x)/range, and a variant of Grubb's outlier test statistic MAD-Grubb and defined as (median-x)/MAD where MAD is the median absolute deviation. MAD-Grub was preferred to Grubb's standard statistic, as it is outlier-resistant, which is beneficial for the detection of outliers at the extreme low end of the distribution, since irrelevant extreme values at the high end of the distribution have little or no influence on the median or the MAD.

### PCR and Sequencing analysis

The coding region of genes of interest was sequenced from genomic DNA extracted from cultured fibroblast cell lines. Intron-located primers were designed using Primer3 v.0.4.0 website [9] for the following genes: *AGA, ADA, ADSL, GAA, ACADM, HPRT1, SURF1, MOCS2, DGUOK, NPC1, NPC2, HEXA *(Additional file 1). PCR products were purified using QIAquick^®^PCR purification Kit (QIAGEN). Dye-terminator cycle sequencing was performed using the BigDye^®^terminator v3.1 cycle sequencing kit (Applied Biosystems, Warrington, UK). Excess dye terminators were removed using Agencourt^®^CleanSeq^® ^(Beckman Coulter, High Wycombe, UK). Samples were run on an ABI PRISM 3130 × l Genetic Analyzer (Applied Biosystem). Sequences were analysed by Mutation Surveyor Local v3.20 (Biogene, Kimbolton UK).

## Results

### Search for a gene expression signature

**Figure 1:**
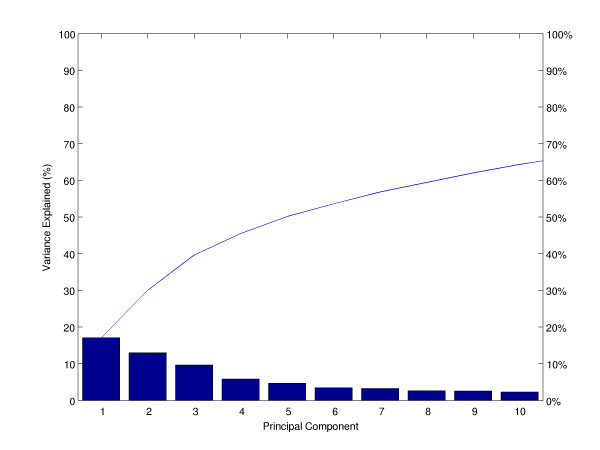
**Principal component analysis of microarray data**. PCA determines the independent axes along which the data exhibits the largest variation. The first ten principal axes/components and their contribution to the overall variance in the data are shown. No single component contributes more than 20% to the overall experimental variation.

**Figure 2:**
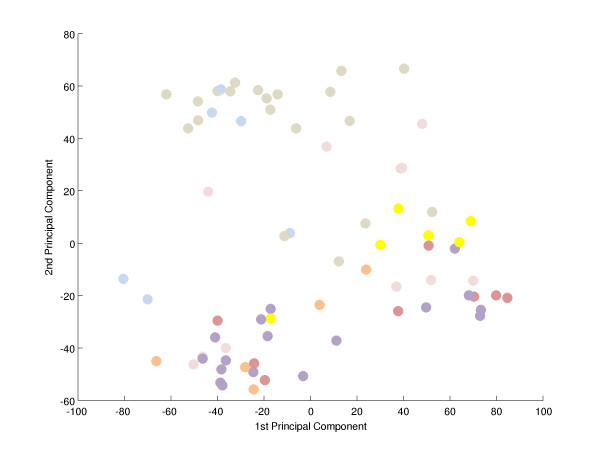
**Principal component analysis-the effect of microarray batch**. An experimental batch effect is apparent. The figure shows a projection of the array measured gene expression profiles of all patients onto the plane spanned by the first two principal components (PCs) that is the two axes along which the data vary the most. Each expression profile (filled circles) is coloured according to microarray batch membership. PC1 separates profiles in the light blue batch (toward the left) from those in the yellow batch (toward the right), while PC2 separates grey (toward the top) from purple, salmon and pale red (towards the bottom; Additional file 2 'Samples').

**Figure 3:**
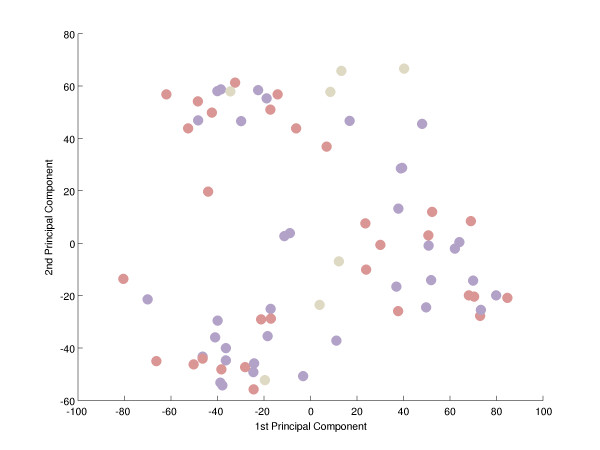
**Principal component analysis-effect of patient gender**. There is no correspondence between PC1 and 2 and patient gender. A projection of all expression profiles onto the plane spanned by the first two PCs is shown. There is no clustering of male (blue) or female (red) arrays, indication that gender does not contribute substantially to gene expression variation. Grey = unknown; see also Additional file 2 'Samples').

**Figure 4:**
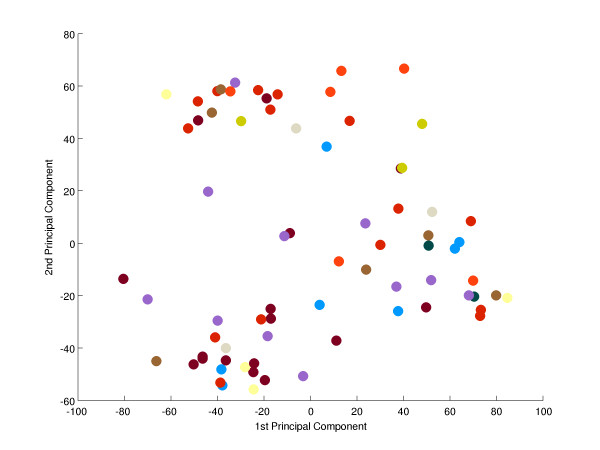
**Principal component analysis-effect of metabolic disease class**. There is no obvious relationship between disease class and the first two PCs. Expression profiles were projected onto the plane spanned by the first two PCs. Each expression profile was coloured according to metabolic disease class (see also Additional file 2 'Samples').

To search for a metabolic signature, principal component analysis (PCA) was applied to identify and quantify independent sources of variation observed in the data. PCA identified no single dominating source of variance (Figure 1). The first and second principle components (PCs) accounted for ~20% and ~15% of overall variance, with the microarray batch experimental variable being the source of these two components of overall variance (Figure 2), as opposed to patient gender or disease category (Figure 3 and 4). Although, the analytical variance introduced by batching the arrays in different experiments was greater than the variation due to biological factors, batching arrays was not a dominant source of variance overall. This was a first indication that transcriptional profiles do not effectively discriminate between different categories of metabolic disease.

**Figure 5:**
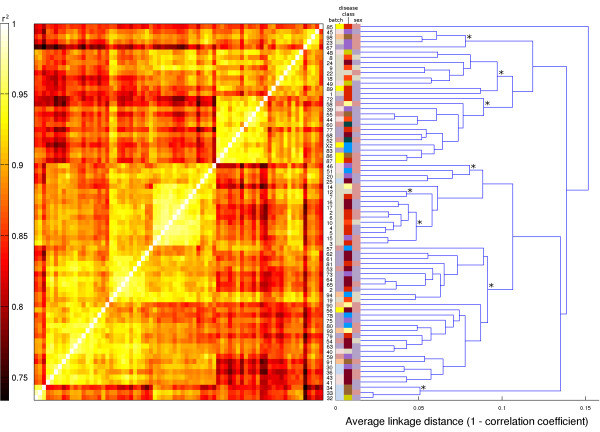
**Heat map visualization of pair-wise correlation coefficients and corresponding hierarchical clustering dendrogram**. There is some batch effect, with arrays from the same batch tending to cluster. For example, there are distinct clusters comprising only arrays from the grey batch. The clusters do not reflect gender or disease class. Heat map visualization of pair-wise correlation coefficients (r^2^; left) between arrays and corresponding hierarchical clustering dendrogram (using average linkage and 1-r^2 ^as the distance metric). The branches corresponding to the eight most distinct non-singleton clusters are labeled by asterisks. A cluster was considered distinct if its inconsistency coefficient (IC) was 1.9 at a depth of up to 5.

Correlation analysis showed that correlation coefficients (r^2^) for any pair of expression profiles ranged from 0.73 to 0.98. While r^2 ^was greater than 0.9 for all pairs of replicate samples (arrays 23 & 67, 17 & 61, 20 & 59, 16 & 62), a sample and its replicate did not typically achieve the maximum r^2 ^(Additional file 2 'FARMS_CCs'). There were other non-identical samples from the same microarray batch for which r^2 ^was greater. Since the replicate of a sample was always processed as part of a different batch, this suggests that there were systematic differences between the microarray batches which were sufficiently large to make replicates appear to be relatively uncorrelated, even though in absolute terms, the correlation between replicates was high. More importantly, this also indicates the absence of disease-dependent systematic effects on gene expression profiles that are large enough to supersede the technical microarray batch effect. This was underscored by the results of applying unsupervised hierarchical clustering to the data (Figure 5). The eight most distinct non-singleton clusters tended to partition the set of samples along microarray batch boundaries, and not according to patient gender or disease class (Additional file 2 'Samples').

### Outlier (NMD) detection

**Figure 6:**
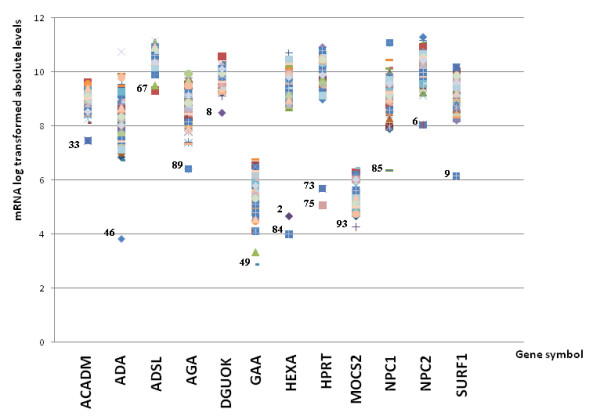
**Genes identified with premature termination codon mutations leading to nonsense mediated decay**. Messenger RNA expression levels for all patients for selected genes are shown. The outliers seen at the bottom of distribution correspond to patients (numbered) with nonsense mediated decay associated mutations. The genes *ACADM, GAA *and *MOCS2 *were excluded from analysis through probe set selection and classified as false negatives.

While metabolic diseases do not appear to result in a specific gene expression profile characteristic of disease-class, we observed that in 14/68 (21%) of the assayed patient fibroblast cell lines, mRNA expression of the gene responsible for the metabolic defect was decreased and well separated from the population (Figure 6). DNA sequencing identified mutations consistent with nonsense-mediated decay (NMD) of the mRNA (Table 2).

**Table 2:** Genes with mutations resulting in premature termination codons and nonsense mediated decay.

Gene	MIM entry	Chip number	Mutation	Predicted effect
ACADM*	607008	33	c.321-324delATTA c.199T > C, [Y67H]	Premature termination

ADA	608958	46	c.350G > A, [W117X], second mutation unknown	Premature termination

ADSL	608222	67	c.7G > C, [A3P] c.578C > T, [R190X]	AA substitution Premature termination

AGA	613228	89	c.788delT	Premature termination

DGUOK	601465	8	c.398C > T, [R105X]	Premature termination

GAA*	606800	49	c.2560C > T, [R854X]	Premature termination

HEXA	606869	2	c.1278-1282insTATC, second mutation unknown	Premature termination

HEXA	606869	84	c.1278-1282insTATC, second mutation unknown	Premature termination

HPRT1	300322	73	g.IVS6+2T > A	3'splice junction (exon insertion)

HPRT1	300322	75	g.IVS7+1G > T	Exon 7 skipping

MOCS2*	603708	93	c.564G > C, [W228C] c.726-727delAA	exon 5 skipping Premature termination

NPC1	607623	85	c.1189C > T, [Q397X]	Premature termination

NPC2	601015	6	c.58G > T, [E20X]	Premature termination

SURF1	185620	9	c.326-327insAT 326-336 del TCTGCCAGCC c.823-842delATCGTGACCTGGTGAAGTC	Premature termination

* Genes excluded from analysis through probe set selection and classified as false negatives

We then determined whether NMD of the disease-causing gene was systematically detectable from the microarray data using outlier statistics. We used two metrics to determine the degree to which a gene expression measurement x constitutes an outlier relative to the patient population: Dixon's Q statistic defined as 2nd-to-minimal-value-x)/range, and a variant of Grubb's outlier test statistic MAD-Grubb defined as median-x)/MAD where MAD is the median absolute deviation. For each metric, we investigated sensitivity and specificity with respect to NMD detection. Since we suspected that the results also depended on the choice of microarray probe-level summarization method, we performed the analysis twice, using Factor analysis for robust microarray summarization (FARMS) or Robust multiarray averaging (RMA) respectively.

Using the FARMS-summarized data (Additional file 2 'FARMS_GoIs), we found that a threshold of Dixon's Q > 0.25 achieved maximum sensitivity. For 11 out of the 14 positive NMD patients, the measurement of a probe set for the specific mutated gene exceeded the threshold. Three patients (33, 49, and 93) were considered false negatives as the probe set or sets for the affected gene (*ACADM, GAA, MOCS2*) were excluded *a priory *due to having been called non-informative during data pre-processing. Therefore, lowering the Dixon's Q threshold did not increase sensitivity and hence, 11/14 was the maximally achievable sensitivity. Using the MAD-Grubb metric, maximum sensitivity was achieved with a threshold of > 4.5.

For the RMA-summarized data (Additional file 2 'RMA_GoIs'), a threshold of Dixon's Q > 0.25 gave a sensitivity of 12 out of 14 positive controls, with the false negatives being 49 and 93. Maximum sensitivity (13/14) was achieved for a threshold of 0.19, which was exceeded for a *MOCS2 *probe set in patient 93. Patient 49 remained a false negative due to the only *GAA *probe set having been excluded during pre-processing. Using the MAD-Grubb metric, maximum sensitivity was achieved with a threshold of > 5.4.

Next, we investigated the specificity of the Dixon's Q and MAD-Grubb outlier metrics. Specifically, we determined, separately for each sample, the fraction of probe sets for which Dixon's Q (or MAD-Grubb) was less than the threshold, while systematically varying the threshold. We estimated the false positive rate (FPR) for a sample as the fraction of probe sets exceeding the threshold. This is a conservative estimate, since for some of the false positive genes polymorphism affecting mRNA expression may be responsible for the decreased expression.

For FARMS-summarized data and a threshold of Dixon's Q > 0.25 (maximum sensitivity 11/14), the false positive rate (FPR) was <0.1% for all samples except sample 34 (FPR < 0.25%. In absolute terms, an FPR of < 0.1% corresponded to, on average, less than 10 probe sets per sample exceeding the threshold from a total of 9,787 probe sets. For the MAD-Grubb threshold of > 4.5 (maximum sensitivity 11/14), the FPR was < 0.9% for most samples. The exceptions were four samples 33, 36, 32, 34 with FPR > 1%, all from the same microarray batch. So, at maximum sensitivity, the FPR for the MAD-Grubb metric was an order of magnitude larger than for Dixon's Q, and MAD-Grubb was more susceptible to microarray batch effects.

For RMA-summarized data and a threshold of Dixon's Q > 0.25 (sensitivity 12/14), the FPR was < 0.25% for all but two samples (34 and 36; FPR > 1%). For MAD-Grubb > 5.4 (maximum sensitivity 13/14), the FPR was < 0.9% for all but four samples (33, 36, 32, 34; FPR > 1%). For Dixon's Q > 0.19 (maximum sensitivity 13/14), the FPR was < 0.5%, again except for samples 34 and 36. Given the total number of 11,753 probe sets in the analysis, an FPR of < 0.25% corresponds to < 30 probe sets.

## Discussion

No evidence of a gene expression signature characteristic of a specific metabolic disorder was found using PCA and hierarchical clustering. Few studies have attempted to characterise mRNA profiles in inherited metabolic disorders. Using microarray-generated expression data, Bozzato et al, compared three fibroblast cell lines from patients with mucolipidoses type IV, an autosomal recessive lysosomal storage disorder, to three control cell lines, and reported differential expression of a number of genes belonging to endosome/lysosome trafficking, lysosome biogenesis, organelle acidification and lipid metabolism [10]. The authors concluded that differential expression of these genes correlated with altered biological processes associated with the disease. Bifsha et al noted down regulation of ubiquitin C-terminal hydrolase (UCH-L1) in eight different lysosomal storage disorder samples [11] suggesting that impairment of the ubiquitin-dependent protein degradation pathway may contribute to increased cell death seen in some of these disorders. We found no clustering of patients with lysosomal disorders that would indicate a gene expression signature. Considerable variation in levels of gene expression between different patient cell lines was found. We have however not defined a 'normal range' for the expression of individual genes as 65/68 of cell lines in the study were derived from patients with a suspected metabolic defect. Gene expression would also be expected to vary under different culture conditions to those used in this study. A proportion of the cohort variation, < 20%, can be ascribed to a batch effect or variation between different experiments. Although variation between experiments is low, this may have been sufficient to mask the identification of a metabolic signature. Non-genetic factors which may contribute to variation in gene expression seen in the population include passage number of the cell lines and differences between cell culture medium batch.

We were able to detect significantly decreased mRNA expression levels of the defective gene relative to the expression range in the study cohort in 11/68 (16%) patients. The low levels of mRNA correlating with premature termination codon (PTC) mutations are consistent with nonsense mediated mRNA decay (NMD), a process which enables the cell to eliminate faulty mRNA that would otherwise translate into aberrant truncated proteins with potential toxic effects for the organism [12-14].

Our results suggest that FARMS-summarization and Informative/Non-Informative (I/NI)-filtering [8] of the array data combined with the Dixon's Q outlier metric provide the best trade-off between sensitivity (> 78%; 11/14 patients) and specificity (> 99.9%) for the purpose of NMD detection. The sensitivity can be improved (> 92%) by using RMA-summarization combined with relatively conservative low-expression threshold filtering and/or using the MAD-Grubb outlier metric. However, this reduces specificity by an order of magnitude which, given the total number of tests performed (~10,000 probe sets), can lead to dozens of genes being identified as potentially undergoing NMD (Additional file 2 'NMD_summary', 'FARMS_NMD', 'RMA_NMD').

Using FARMS-summarization and I/NI-filtering of the array data, three false negatives were identified with NMD-associated mutations in *ACADM*, *MOCS2 *and *GAA*. These three genes were identified as outliers and true positives when Dixon's Q outlier metric was applied to the unfiltered data. This represents a limitation of the assay as only genes with significant levels of expression in fibroblasts were included in the analysis in order to maximize specificity. As a result, disease associated genes expressed at a low level or not expressed at all in fibroblasts will be excluded from the analysis.

For FARMS-summarized data and a threshold of Dixon's Q > 0.25 (maximum sensitivity 11/14 patients), the false positive rate (FPR) of < 0.1% corresponded on average, to less than 10 of 9,787 probe sets per sample exceeding the threshold for detection as an outlier. For example, for cell line 73 with a confirmed deficiency of the enzyme HPRT due to the mutation, *HPRTg.IVS6+2T > A *(Table 2), genes *LPP, SKIL, ZNF281, PDLIM7, COL1A2*, and *AMIGO2 *were detected as outliers in addition to *HPRT1 *(Table 3). For cell line 75, also with a confirmed deficiency of enzyme HPRT, with mutation, *HPRTg.IVS7+1G > T *(Table 2), genes *SF1*, *MARS*, *TCEA2*, *ANKRD13A*, and *PHF13 *(Table 3) were detected as outliers in addition to *HPRT1*. The outlier genes identified in the two HPRT deficient patients were different and none of these genes to our knowledge are disease associated. It is also possible that low levels of mRNA detected as false positives may be the result of promoter variants or asymptomatic heterozygous PTC mutations; however this was not investigated. Clinical phenotypes provide guidance to limit the number of candidate disease-associated genes for further investigation.

**Table 3:** Genes identified as false positives (FP) after FARMS-summarization and I/NI-filtering of the data combined with Dixon's Q outlier metric in true positive (TP) patients.

Patient identifier	TP NMD gene symbol	Num of FP	Gene symbol
85	NPC1	12	LBH, SGCD, SLC1A4, PHF10, ID4, NRAS, S100A4, SHMT2, SETBP1, BACE1, LONRF1, CXXC5

6	NPC2	0	-

2	HEXA	0	-

84	HEXA	2	LRCH2, CHCHD7

89	AGA	7	SSR2, FAR1, NOL12, NAV1, TRIOBP, SCCPDH, HSP90B1

75	HPRT1	5	SF1, MARS, TCEA2, ANKRD13A, PHF13

73	HPRT1	6	LPP, SKIL, ZNF281, PDLIM7, COL1A2, AMIGO2, STUB1, CD44, RAD23A, ZNF598, PCGF1, EMP1, FXYD5

67	ADSL	21	STEAP1, MAP4K4, TMEM22, ASCC2, PDLIM4, HGS, ACAP3, PNKP, EMP3, LMNA, FLII, C11orf68, FLI10357

46	ADA	7	IL1R1, APLP2, SLC30A1, ANKRD57, APLP2, SOCS2, RECK

8	DGUOK	1	TMEM47

9	SURF1	1	CIRBP

There are more than 300 different inherited metabolic diseases [4,15]. Nonsense and frameshift mutations generating PTCs account for approximately one third of mutations in human genetic diseases [16]. In our study, the defective gene could be identified in 16% of patients with an IMD. Fibroblast cell cultures are often established in patients with suspected familial metabolic disorders where initial screening tests have proven uninformative. It is in this group of patients where gene expression may contribute significantly to shortcutting the diagnostic cascade.

## Conclusion

In this study, we investigated whether microarray gene expression profiling of cultured fibroblasts could identify the metabolic defect in 68 patients with proven or suspected inherited metabolic diseases. Using this approach, we were able to identify the defective gene in 16% of patients irrespective of the underlying metabolic defect. There are a number of emerging technologies which will find application in the routine diagnosis of genetic disorders. These include targeted re-sequencing chips aimed at specific groups of disorders [17] and massively parallel next generation sequencing, which is orders of magnitude more expensive than gene expression profiling. We suggest that due to the relatively low cost of microarray gene expression profiling, this technology has a role to play in the diagnosis of genetic disorders where first-line screening tests are uninformative.

## Competing interests

The authors declare that they have no competing interests.

## Authors' contributions

The study was designed by AMM, MAH and DP. A first draft of the manuscript was prepared by MAH, RS and AMM. MAH was responsible for the laboratory aspects of the study. RS analysed the microarray data with contributions from MAH and AMM. Clinical information and samples were provided by MPC, JWT, and AF. Access to microarray facilities and technical support were provided by BDY, DP, and TC.

All authors read and approved the final manuscript.

## Supplementary Material

Additional file 1 **Table S1**. Primers and PCR conditions Click here for file

Additional file 2 **Excel spread sheet containing the following data**. Sample/Patient annotation, **FARMS_CCs**: Pair-wise correlation coefficients between gene expression profiles for all samples, based on FARMS data, **FARMS_GoIs**: Annotation and outlier test for metabolic genes of interest using FARMS expression values and I/NI filtering, **RMA_GoIs**: Annotation and outlier test for metabolic genes of interest using RMA expression values and 100 as expression cut-off, **FARMS_NMD**: NMD candidates based on FARMS data, **RMA_NMD**: NMD candidates based on RMA data, **NMD_summary**: Number of probe sets exceeding Dixon Q threshold. Click here for file
