# Supplementary material for: The diagnosis of inherited metabolic diseases by microarray gene expression profiling
Source: Orphanet J Rare Dis. 2010 Dec 1;5:34. doi: 10.1186/1750-1172-5-34 (PMC3009951; doi:10.1186/1750-1172-5-34)
Supplement: Additional file 1 — Table S1. Primers and PCR conditions [file 1750-1172-5-34-S1.DOC]

**Table S1**: Primers and PCR conditions.
